# Electrophysiological and Morphological Properties of α and γ Motoneurons in the Rat Trigeminal Motor Nucleus

**DOI:** 10.3389/fncel.2018.00009

**Published:** 2018-01-24

**Authors:** Kayo Nishimura, Masahiro Ohta, Mitsuru Saito, Yukako Morita-Isogai, Hajime Sato, Eriko Kuramoto, Dong Xu Yin, Yoshinobu Maeda, Takeshi Kaneko, Takashi Yamashiro, Kenji Takada, Seog Bae Oh, Hiroki Toyoda, Youngnam Kang

**Affiliations:** ^1^Department of Neuroscience and Oral Physiology, Osaka University Graduate School of Dentistry, Suita, Japan; ^2^Department of Orthodontics and Dentofacial Orthopedics, Osaka University Graduate School of Dentistry, Suita, Japan; ^3^Department of Removable Prosthodontics, Osaka University Graduate School of Dentistry, Suita, Japan; ^4^Department of Oral Physiology, Graduate School of Medical and Dental Sciences, Kagoshima University, Kagoshima, Japan; ^5^Department of Oral Anatomy and Cell Biology, Graduate School of Medical and Dental Sciences, Kagoshima University, Kagoshima, Japan; ^6^Department of Morphological Brain Science, Graduate School of Medicine, Kyoto University, Kyoto, Japan; ^7^Department of Neurobiology and Physiology, School of Dentistry, Seoul National University, Seoul, South Korea

**Keywords:** α-motoneuron, γ-motoneuron, trigeminal motor nucleus, NeuN, electrophysiology

## Abstract

The muscle contraction during voluntary movement is regulated by activities of α- and γ-motoneurons (αMNs and γMNs, respectively). The tension of jaw-closing muscles can be finely tuned over a wide range. This excellent function is likely to be achieved by the specific populations of αMNs innervating jaw-closing muscles. Indeed, we have recently demonstrated that in the rat dorsolateral trigeminal motor nucleus (dl-TMN), the size distribution of αMNs was bimodal and the population of smaller αMNs showed a size distribution similar to that of γMNs, by immunohistochemically identifying αMNs and γMNs based on the expressions of estrogen-related receptor gamma (Err3) and neuronal DNA binding protein NeuN together with ChAT. This finding suggests the presence of αMNs as small as γMNs. However, differences in the electrophysiological membrane properties between αMNs and γMNs remain unknown also in the dl-TMN. Therefore, in the present study, we studied the electrophysiological membrane properties of MNs in the dl-TMN of infant rats at postnatal days 7–12 together with their morphological properties using whole-cell current-clamp recordings followed by immunohistochemical staining with an anti-NeuN and anti-ChAT antibodies. We found that the ChAT-positive and NeuN-positive αMNs were divided into two subclasses: the first one had a larger cell body and displayed a 4-aminopyridine (4-AP)-sensitive current while the second one had a smaller cell body and displayed a less prominent 4-AP-sensitive current and a low-threshold spike, suitable for their orderly recruitment. We finally found that γMNs showing ChAT-positive and NeuN-negative immunoreactivities had smaller cell bodies and displayed an afterdepolarization mediated by flufenamate-sensitive cation current. It is suggested that these electrophysiological and morphological features of MNs in the dl-TMN are well correlated with the precise control of occlusion.

## Introduction

The muscle contraction during voluntary movement is precisely regulated by activities of α- and γ-motoneurons (αMNs and γMNs, respectively), and the activations of αMNs and γMNs occur almost simultaneously (Phillps, 1969; Matthews, 1972). This phenomenon was termed as α–γ coactivation, which is thought to be necessary to compensate for mechanical unloading caused by the extrafusal shortening so that the stretch receptors contained in the muscle spindle remains sensitive (Desmedt, 1983). The α–γ coactivation also plays an important role in voluntary isometric contraction of not only human lumbrical muscles (Vallbo, 1971) but also jaw-closing muscles during the slow jaw-closing phase. It is known that the spindle Ia impulse is involved in producing 30–40% of the isometric contraction of hand and leg muscles (Gandevia et al., 1990; Macefield et al., 1993). Consistent with these reports, we previously showed that the muscle spindle Ia activity caused by γMN activity is involved in precisely regulating isometric contraction of masseter muscles (Tsukiboshi et al., 2012), during which αMNs are likely to be orderly recruited (Okamoto et al., 2016). Thus, activities of γMNs play a crucial role in the isometric contraction. However, little is known about the firing pattern or excitability of γMNs compared to αMNs, and the intrinsic membrane properties of γMNs have hardly been investigated for quite a long time even in *in vitro* preparations.

Recently, molecular markers that distinguish between αMNs and γMNs were identified. Estrogen-related receptor gamma (Err3) is one of nuclear receptors, and is involved in the differentiation of γMNs (Friese et al., 2009). It has been demonstrated that in a mouse spinal motor nucleus, Err3 is expressed at high levels in γMNs but not αMNs, while the neuronal DNA binding protein NeuN is expressed at high levels in αMNs but not in γMNs (Friese et al., 2009). We have also reported that MNs in the dorsolateral trigeminal motor nucleus (dl-TMN) are composed of 65% αMNs (Err3-negative/NeuN-positive MNs) and 35% γMNs (Err3-positive/NeuN-negative MNs). The size distribution of αMNs was bimodal while that of γMNs was almost the same as that of the population of small αMNs, suggesting the presence of αMNs as small as γMNs in the dl-TMN (Morita-Isogai et al., 2017). In the present study, we aimed to elucidate the electrophysiological and morphological properties of αMNs and γMNs in the dl-TMN using whole-cell current-clamp recordings together with immunohistochemical (IH) stainings with anti-Err3 and anti-NeuN antibodies to identify whether the recorded neuron is αMN or γMN.

## Materials and Methods

### Ethical Approval

The experimental protocol was approved by the animal ethics committees of the Osaka University Graduate School of Dentistry for the care and use of laboratory animals, and the experiment was performed in accordance with the relevant guidelines.

### Slice Preparation

The procedure for slice preparation was the same as that in the previous study (Okamoto et al., 2016). Using Wistar rats of both sexes at postnatal days (PND) 7–12 (Nihon Dobutsu, Osaka, Japan), coronal sections of 250 μm thickness including the dl-TMN were cut.

### Whole-Cell Recording from MNs in the dl-TMN

The electrophysiological studies were performed on the MNs in the dl-TMN. Axopatch 200B (MDS Analytical Technologies, Sunnyvale, CA, United States) was used for whole-cell patch-clamp recordings. The artificial cerebrospinal fluid (aCSF) solution had the following composition (mM): 123 NaCl, 1.8 KCl, 2.5 CaCl_2_, 1.3 MgCl_2_, 26 NaHCO_3_, 1.2 KH_2_PO_4_, and 10 glucose, bubbled with mixture of 95% O_2_–5% CO_2_. The internal solution had the following composition (mM): 118 K-gluconate, 8 KCl, 20 NaCl, 0.5 MgCl_2_, 2 ATP-Na_2_, 0.3 GTP-Na_3_, 10 HEPES, 0.1 EGTA, and 10 biocytin; the pH was adjusted to 7.3 with KOH. The patch pipettes had a DC resistance of 4–5 MΩ when filled with the internal solution. The membrane potential values given in the text were corrected for the junction potential between the internal solution (negative) and the standard extracellular solution (10 mV). All recordings were made at room temperature (20–24°C). The sealing resistance was usually more than 10 GΩ. Whole-cell voltages were low-pass filtered at 2 kHz (4-pole Bessel filter), digitized at a sampling rate of 10 kHz (Digidata 1322A, MDS Analytical Technologies). Under the current-clamp condition, depolarizing and hyperpolarizing current pulses were applied every 30 s. To dissect transiently inactivating components, such as low-threshold spike (LTS) or the responses mediated by A-like K^+^ current, depolarizing current pulses were applied both approximately at -85 and -65 mV, at which voltage-dependent inactivation can largely be removed and progress, respectively. To measure the relationship between current intensities and the first instantaneous or steady-state firing frequencies, depolarizing current pulses applied at -80 mV were linearly increased in amplitude. The input resistance was calculated from the linear portion of current–voltage (*I–V*) relationships.

### Fluorescence Immunohistochemistry

**FIGURE 1:**
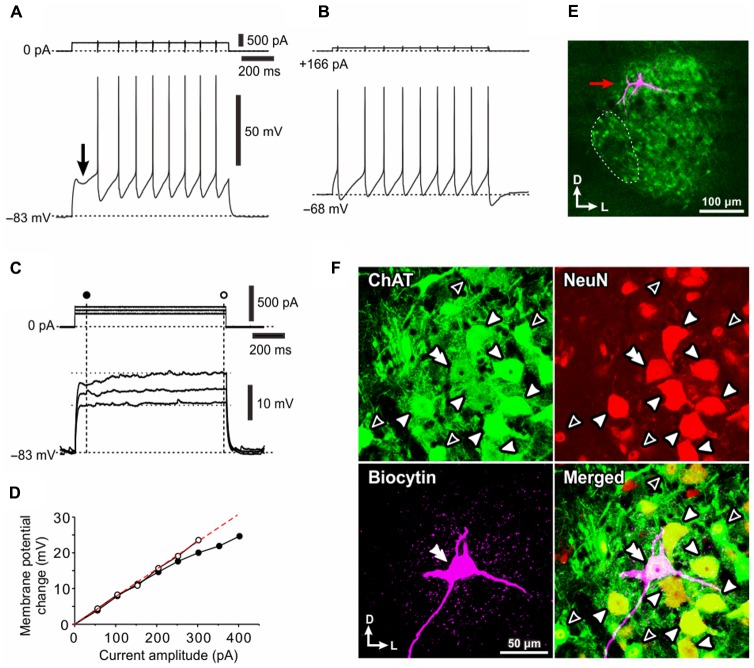
Electrophysiological membrane properties of Type I αMNs. **(A,B)** Spike trains evoked in response to depolarizing current pulses applied in a neuron classified as Type I αMN at a resting membrane potential of –83 mV **(A)** and at a depolarized baseline potential of –68 mV brought about by 166 pA DC current injection **(B)**. An arrow indicates a hyperpolarizing notch that caused a delay in the generation of the first spike **(A)**. **(C)** Subthreshold membrane potential responses to depolarizing current pulses applied at the resting membrane potential of –83 mV. **(D)** A relationship between the amplitude of depolarizing current pulses and the membrane potential change measured 60 ms after the pulse onset (**C**, filled circles) and that measured 10 ms before the pulse offset (**D**, open circles). **(E)** A fluorescence image of the recorded neuron (red arrow) labeled with biocytin located among ChAT(+) MNs in the dl-TMN. The area encircled with the dotted line is the accessory-TMN. **(F)** Confocal images showing immunoreactivity for ChAT (green), NeuN (red), and biocytin (pink). Merged, a merged fluorescence image. A double filled arrowhead indicates the biocytin-labeled ChAT(+) and NeuN(+/N, +/C) αMN. Filled arrowheads indicate ChAT(+) and NeuN(+N, +C) αMNs. Open arrowheads indicate ChAT (+) and NeuN(+/N, –/C) MNs.

After whole-cell recordings, brain slices including the dl-TMN were fixed in solution containing 4% formaldehyde and 75% saturated picric acid at pH 7.2. After washout of 0.1 M PBS, brain slices were frozen overnight in PBS containing 30% sucrose. After washout of 0.1 M PBS, brain slices were incubated overnight at room temperature in PBS containing the following: 0.3% Triton X-100, 0.12% aaa-carrageenan, 0.02% sodium azide, and 0.3% normal donkey serum (PBS-XCD) with a mixture of 1:100-diluted goat anti-ChAT antiserum (AB144P; Millipore Bioscience Research Reagents, Billerica, MA, United States), 1 μg/ml of mouse anti-NeuN antibody (MAB377; Millipore Bioscience Research Reagents) and 2 mg/ml Alexa Fluor 488-conjugated avidin (A-21370; Invitrogen, Carlsbad, CA, United States). For the immunostaining of NeuN and ChAT, the brain slices were then incubated for 1 h with 1 μg/ml TSA Cyanine 3 (Cy3) System (PerkinElmer, Waltham, MA, United States) and 2 μg/ml Alexa Fluor 647-conjugated donkey anti-goat IgG antibody (Invitrogen). The sections were observed with a confocal laser-scanning microscope (LSM510; Zeiss, Oberkochen, Germany). Alexa Fluor 647, Cy3, and Alexa Fluor 488 were excited with 633, 543, and 488 nm laser beams, and observed through 660–788, 560–615, and 505–530 nm emission filters, respectively. The digital images were captured by using a software (LSM510; Zeiss). The cross-sectional area of the MNs was measured at a plane intersecting the nucleoli of the MNs using Photoshop CS6 (Adobe Systems Software), and the major and minor axis lengths of an MN were measured. Morphological analyses of dendritic arborization of biocytin labeled MNs were not possible in the present study because the somata of biocytin labeled MNs were located either near the surface or between the top and bottom surfaces of the slice preparation, depending on which dendritic arborization may be either largely severed (e.g., **Figure 1E**) or relatively preserved (e.g., **Figure 3E**).

### Data Analysis

Statistical analysis was performed using STATISTICA10J (StatSoft). Numerical data were expressed as the mean ± SD. The statistical significance was assessed using one-way ANOVA (^†^) followed by Fisher’s protected least significant difference *post hoc* test, unpaired (^∗^) and paired (^∗∗^) Student’s *t*-test and Wilk’s lambda (#). Student’s *t*-test was used when the data showed the normal distribution as confirmed by K–S test. *p* < 0.05 was considered statistically significant.

## Results

First, we recorded from relatively large-sized neurons in the dl-TMN, which were classified into the two groups based on the electrophysiological membrane properties: one classified as Type I was characterized by a prominent A-type K^+^ current (*I*_KA_) and the other classified as Type II was characterized by *I*_KA_ and LTS. Both the Type I and II neurons were immunopositive for ChAT and NeuN, and were thereby identified as αMNs. Second, we recorded from relatively small-sized neurons in the dl-TMN, which were classified as either Type II αMNs or gMNs. Those MNs classified as gMNs were characterized by the afterdepolarization (ADP) which is mediated by flufenamic acid-sensitive cationic current, and were immunopositive for ChAT but not prominently for NeuN.

### Electrophysiological and Morphological Properties of Type I αMNs

When a depolarizing current pulse was injected into a neuron in the TMN at the resting membrane potential of -83 mV (**Figure 1A**), the generation of the first spike was largely delayed from the onset of the current pulse due to a preceding hyperpolarizing notch (**Figure 1A**, arrow). In contrast, when a depolarizing current pulse was injected at a depolarized baseline potential of -68 mV which was brought about by a positive DC current injection of 166 pA (**Figure 1B**, upper), the delay was markedly reduced (**Figure 1B**, bottom). Furthermore, the relationship between the amplitude of depolarizing current pulses and the membrane potential changes measured 60 ms after the pulse onset (**Figure 1C**, filled circle) displayed a non-linear relationship that reflects the outwardly rectifying nature of some K^+^ current (**Figure 1D**, filled circles) whereas that measured 10 ms before the pulse offset (**Figure 1C**, open circle) was almost linear (**Figure 1D**, open circles). These results suggest that the delayed spike generation is caused by activation of an early transient outward K^+^ current.

After whole-cell current-clamp recordings, we performed triple immunofluorescence staining for biocytin, ChAT, and NeuN in the TMN (**Figures 1E,F**). ChAT immunoreactivity was detected in many neurons within the TMN, which could be divided into the dl-TMN and ventromedial- or accessory-TMN (encircled by an interrupted line), the latter of which is the region where jaw-opening MNs are localized (Mizuno et al., 1975; Sengul and Watson, 2012) (**Figure 1E**). Biocytin labeling revealed that the recorded neuron was located in the dorsal part of the TMN (**Figure 1E**, red arrow). The triple immunofluorescence staining revealed that the biocytin-labeled neuron was immunopositive for ChAT, namely ChAT(+), and immunopositive for NeuN not only in the nucleus but also in the cytoplasm, namely NeuN(+/N, +/C) (**Figure 1F**, double filled arrowhead), indicating that the recorded neuron can be identified as an αMN. Thus, we classified those αMNs that are characterized by an early transient outward K^+^ current as Type I αMNs The recorded Type I αMN was intermingled with ChAT(+) MNs (open arrowheads) that are immunopositive for NeuN only in their nuclei but not in the cytoplasm, namely NeuN(+/N, -/C), as well as ChAT(+) and NeuN(+/N, +/C) αMNs (filled arrowheads) in the dl-TMN (**Figure 1F**) (see section “Discussion”).

### Involvement of 4-AP Sensitive K^+^ Current in the Delayed Activation of Type I αMNs

**FIGURE 2:**
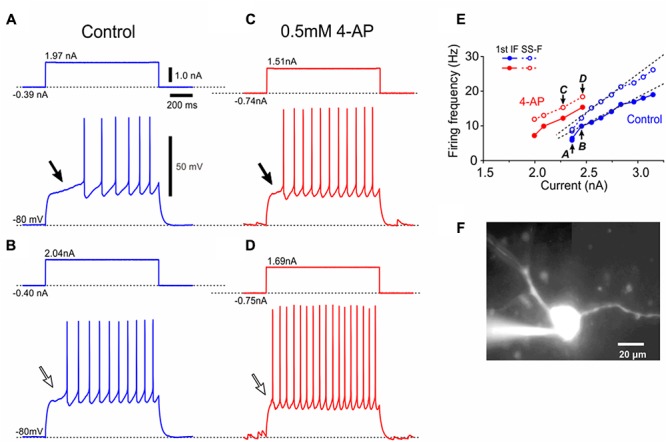
A Type I presumed-αMN displays 4-AP sensitive K^+^ currents. **(A–D)** Spike trains evoked in a neuron in response to depolarizing current pulses applied at –80 mV brought about by negative DC current injections before **(A,B)** and after application of 0.5 mM 4-AP **(C,D)**. The generation of the first spike was largely delayed (**A,B**, lower traces, compare the portions of the traces indicated with filled and open arrows). 4-AP largely decreased the delay or almost abolished it (**C,D**, lower traces, compare the portions of the traces indicated with filled and open arrows). **(E)** The relationship between the current amplitudes and the first IFs (the inverse of the interval between the first and second spikes) or the SS-Fs (the mean firing frequency during the latter half of the current pulses) in spike trains before and after application of 4-AP (blue and red filled or open circles, respectively). The *I*–*F* slope for the first IF, 19 Hz/nA. The *I*–*F* slope for the SS-F, 24 Hz/nA. The plots denoted by arrows with letters of A–D represent the relationships obtained from the spike trains evoked by the corresponding currents shown in **(A–D)**. **(F)** A lucifer yellow image of the recorded Type I presumed-αMN which has a relatively larger multipolar soma.

Because it has been demonstrated that a similar delayed spike generation was abolished after application of 4-aminopyridine (4-AP) in many neurons (Chandler et al., 1994; Saito et al., 2006), we also examined the effects of 4-AP on the delayed spike generation. When depolarizing current pulses were applied at -80 mV in a neuron (**Figures 2A,B**, upper), the generation of the first spike was largely delayed from the onset of the current pulse (**Figures 2A,B**, bottom, filled and open arrows), which characterizes the recorded neuron as a Type I αMN. Following application of 0.5 mM 4-AP, the delay became smaller or was almost abolished (**Figures 2C,D**, bottom, filled and open arrows) in the responses to injections of current pulses with the same amplitudes applied at more negative holding currents which brought the baseline membrane potential back to the original level.

The current–frequency (*I*–*F*) plot revealed that the relationship between current amplitudes and the first instantaneous frequencies [first instantaneous frequency (IF); the inverse of the interval between the first and second spikes] was almost linear (**Figure 2E**, blue filled circles), and so too was that between current amplitudes and the steady-state frequencies (SS-F; mean firing frequency during the latter half of a current pulse) (**Figure 2E**, blue open circles). In eight Type I αMNs either identified immunohistochemically (*n* = 4) or presumed electrophysiologically based on the delayed spiking pattern (*n* = 4), the first IF was invariably smaller than the SS-IF regardless of the intensity of current pulses, and the slope of the *I*–*F* plot for the first IF (23 ± 10 Hz/nA) (**Figure 2E**, blue filled circles) was significantly (^∗∗^*p* < 0.04, *n* = 8) smaller than that for the SS-F (30 ± 14 Hz/nA) (**Figure 2E**, blue open circles). Following application of 4-AP, the relationship between current amplitudes and the first IFs was shifted in the leftward direction, and so too was that between current amplitudes and the SS-Fs (**Figure 2E**). These results indicate that 4-AP sensitive K^+^ currents are involved in the delayed generation of the first spike and compromised the ability of neurons to generate high firing rates. A lucifer yellow image revealed that this neuron has a relatively larger multipolar soma (**Figure 2F**). Thus, Type I αMNs or the Type I presumed-αMNs were characterized by the late spiking and depressed firing frequency, both of which were caused by 4-AP sensitive early outward transient K^+^ current.

### Electrophysiological and Morphological Properties of Type II αMNs

**FIGURE 3:**
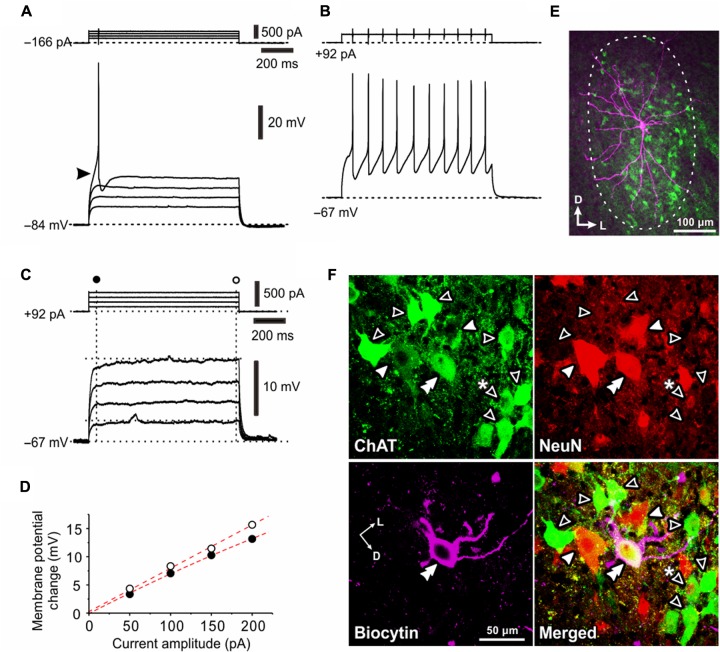
Electrophysiological membrane properties of Type II αMNs. **(A,B)** Spike responses evoked by injection of depolarizing current pulses applied in a neuron classified as Type II αMN at –84 and –67 mV, respectively, which were brought about by a negative and positive DC current injections of –166 pA **(A)** and 92 pA**(B)**, respectively. An arrowhead indicates an LTS-like response **(A)**. **(C)** Subthreshold membrane potential responses to depolarizing current pulses applied in the same Type II αMN at –67 mV. **(D)** A relationship between the amplitude of depolarizing current pulse and the membrane potential change measured 50 ms after the pulse onset (**C**, filled circles) and that measured 10 ms before the pulse offset (**C**, open circles). **(E)** A fluorescence image of the recorded neuron labeled with biocytin (pink) located among ChAT(+; green) MNs in the center of the TMN, showing multiple primary dendrites extending almost the whole TMN. **(F)** Confocal images showing immunoreactivity for ChAT (green), NeuN (red), and biocytin (pink). Merged, a merged fluorescence image. A double filled arrowhead indicates a biocytin-labeled ChAT(+) and NeuN(+/N, +/C) αMN. Filled arrowheads indicate ChAT(+) and NeuN(+/N, +/C) αMNs. Open arrowheads indicate ChAT(+) and NeuN(–/N, –/C) non-α type MNs. Open arrowhead with asterisk indicates a ChAT(+) and NeuN(+/N, –/C) non-α type MNs.

When depolarizing current pulses were injected in a neuron at -84 mV that was brought about by injection of a negative DC current of -166 pA (**Figure 3A**, upper), an LTS-like response was triggered at around -55 mV, from which an action potential was generated (**Figure 3A**, bottom, arrowhead). In contrast, when depolarizing current pulses were injected at -67 mV which was brought about by injection of a positive DC current of +92 pA at the resting membrane potential of -74 mV (**Figure 3B**, upper), the spike triggering was not as fast as that from the LTS-like response, but rather appeared to be slightly delayed (**Figure 3B**, bottom). Indeed, the *I–V* relationship of the subthreshold membrane potential responses to the depolarizing current pulses (**Figure 3D**, filled circles) measured 50 ms after the pulse onset (**Figure 3C**, filled circle) displayed a non-linear relationship that reflects the outwardly rectifying nature of some K^+^ current whereas that measured 10 ms before the pulse offset (**Figure 3C**, open circle) was almost linear (**Figure 3D**, open circles).

After the recordings, we performed triple immunofluorescence staining for biocytin, ChAT and NeuN in the TMN (**Figures 3E,F**). Biocytin immunoreactivity revealed that the recorded neuron had multiple primary dendrites and was located in the middle portion of the dl-TMN (**Figure 3E**). The triple immunofluorescence staining revealed that the biocytin-labeled neuron was ChAT(+) and NeuN(+/N, +/C) (**Figure 3F**, double filled arrowhead). Around the recorded neuron, NeuN(+/N, +/C) αMNs (filled arrowheads) and NeuN(+/N, -/C) MNs (open arrowheads) were intermingled in the dl-TMN (**Figure 3F**). We classified those αMNs that were characterized by *I*_KA_ and LTS as Type II. Unlike the observations as shown in **Figure 1E**, there were many ChAT(+) neurons that were completely immunonegative for NeuN at any *Z*-axis levels ranging between the top and bottom surfaces of their cell bodies, namely NeuN(-/N, -/C), except the one ChAT(+) and NeuN(+/N, -/C) MN (**Figure 3F**, open arrowhead with asterisk) similar to those seen in **Figure 1E** (see section “Discussion”).

**FIGURE 4:**
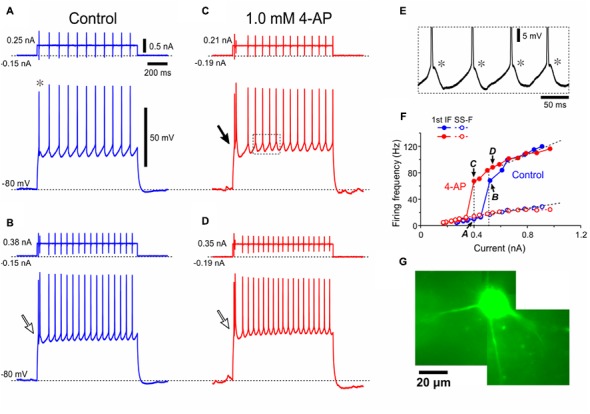
A Type II presumed-αMN displays 4-AP sensitive K^+^ currents and LTS. **(A–D)** Spike trains evoked in response to depolarizing current pulses applied in a Type II presumed-αMN at –80 mV which was brought about by negative DC current injections before **(A,B)** and after application of 1 mM 4-AP **(C,D)**. Note the smaller amplitude of the first spike (asterisk). Open and filled arrows indicate bursts caused by LTS (**B–D**, filled and open arrows). **(E)** The enlargement of the portion of the trace surrounded by a rectangle in **(C)** showing spike-ADPs (asterisks). **(F)** Relationships between the current amplitudes and the first IFs or the SS-Fs before and after application of 4-AP (blue and red filled or open circles, respectively). The *I*–*F* slope for the first IF, 52 Hz/nA. The *I*–*F* slope for the SS-F, 28 Hz/nA. The plots denoted by arrows with letters of A–D represent the relationships obtained from the spike trains evoked by the corresponding currents shown in **(A–D)**. **(G)** A lucifer yellow image of the recorded Type II presumed-αMN which has the multipolar soma.

To further dissect the properties of Type II αMNs, spike trains were induced by applying depolarizing current pulses with various amplitudes at -80 mV in a Type II αMN as characterized by a smaller amplitude of the first spike reflecting an involvement of *I*_KA_ in decreasing the spike amplitude (**Figure 4A**, ^∗^) and a burst caused by LTS (**Figures 4B–D**). The enlargement of the portion of the trace surrounded by a rectangle in **Figure 4C** clearly revealed that each spike was followed by an ADP (spike-ADP) (**Figure 4E**). This result is consistent with the observation made in the TMN of adult rats (Kobayashi et al., 1997). As illustrated in the relationship between current amplitudes and firing frequencies, the SS-F linearly increased with increases in the current amplitude (**Figure 4F**, blue open circles) whereas the first IF displayed a stepwise increase due to a generation of burst by LTS at a certain current intensity, below or above which the first IF displayed linear increases with increases in the current amplitude (**Figure 4F**, blue filled circles). In 11 Type II αMNs either identified immunohistochemically (*n* = 4) or presumed electrophysiologically based on the bursting spike pattern (*n* = 7), the first IF was invariably larger than the SS-IF regardless of the intensity of current pulses when measured after LTS was generated to trigger spikes (**Figure 4F**, blue filled and open circles), in contrast to Type I αMNs. The slope of the *I*–*F* plot for the first IF (118 ± 62 Hz/nA) (**Figure 4F**, blue filled circles) was significantly (^∗∗^*p* < 0.002, *n* = 11) larger than that for the SS-F (39 ± 16 Hz/nA) (**Figure 4F**, blue open circles), also in contrast to Type I αMNs. Following application of 4-AP, the relationship between current amplitudes and the first IFs was shifted in the left direction (**Figure 4F**, red filled circles) but leaving the relationship between current amplitudes and the SS-Fs almost unchanged (**Figure 4F**, red open circles). These results indicate that at the onset of current pulses Type II αMNs can display burst firings mediated by LTS that is modulated by 4-AP sensitive K^+^ currents. A lucifer yellow image revealed that the Type II αMN has a multipolar soma with at least four primary dendrites (**Figure 4G**).

### Electrophysiological and Morphological Properties of γMNs

**FIGURE 5:**
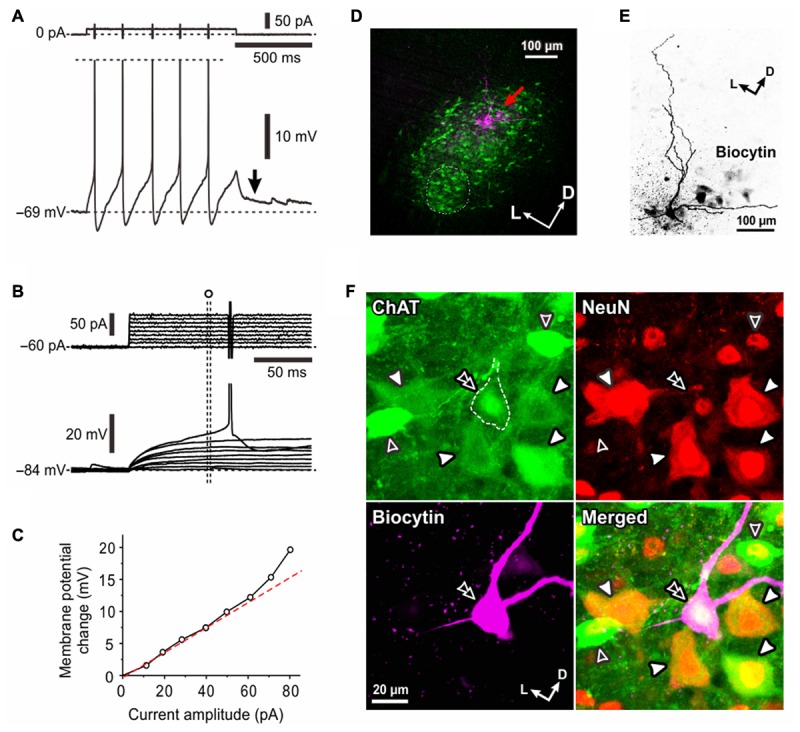
A Electrophysiological membrane properties of γMNs. **(A)** A spike train evoked in response to a depolarizing current pulse applied in a neuron classified as γMN at the resting membrane potential of –69 mV. An arrow indicates a pulse-ADP observed after the pulse offset. Spikes are truncated. **(B)** Subthreshold membrane potential responses to depolarizing current pulses applied in the same γMN at –84 mV which was brought about by a negative DC current injection of –60 pA. **(C)** A relationship between the amplitude of depolarizing current pulse and the membrane potential change from the baseline potential measured at 67–70 ms after the pulse onset (open circles). **(D)** A fluorescence image of the recorded neuron (red arrow) labeled with biocytin located among ChAT(+, green) MNs in the center of the dl-TMN. The area encircled with the dotted line is the ventromedial-TMN. **(E)** Biocytin labeling of the recorded neuron revealed that the recorded neuron displayed sparse arborizations of primary dendrites. **(F)** Confocal images showing immunoreactivity for ChAT (green), NeuN (red), and biocytin (pink). Merged, A merged fluorescence image. A double open arrowhead indicates a biocytin-labeled ChAT (+) and NeuN(+/N, –/C) γMN. Filled arrowheads indicate ChAT(+) and NeuN(+/N, +/C) aMNs. Open arrowheads indicate ChAT(+) and NeuN(+/N, –/C) γMNs.

Next, we recorded from relatively small-sized MNs in the dl-TMN. When a spike train was evoked by injection of a depolarizing current pulse in a neuron at the resting membrane potential (-69 mV), a pulse ADP (pulse-ADP) was induced after the pulse offset and lasted for more than 500 ms (**Figure 5A**, arrow). In the same neuron, subthreshold responses were examined at -84 mV which was brought about by a negative DC current injection of -60 pA (**Figure 5B**). With an increase in the amplitude of depolarizing current pulses, the amplitude of subthreshold membrane potential responses super-linearly increased (**Figure 5B**) as revealed by the relationship between the depolarizing current pulse amplitudes and the changes in membrane potential responses at 67–70 ms after the pulse onset (**Figure 5C**). This finding indicates the existence of a persistent inward current in this neuron. When the pulse-ADP was observed in an MN, the persistent inward current was observed invariably together with the pulse-ADP. In six γMNs identified immunohistochemically, the slope of the *I*–*F* plot for the first IF (252 ± 116 Hz/nA) was significantly (^∗∗^*p* < 0.007) larger than that for the SS-F (143 ± 82 Hz/nA) (figure not shown).

After the whole-cell current recording, we performed triple immunofluorescence staining for biocytin, ChAT and NeuN (**Figures 5D–F**). Biocytin labeling revealed that the recorded neuron was located in the dl-TMN and displayed sparse arborizations of primary dendrites (**Figures 5D,E**). The triple immunofluorescence staining revealed that the biocytin-labeled neuron was ChAT(+) and NeuN(+/N, -/C) (**Figure 5F**, double open arrowhead). This NeuN-immunoreactivity was distinct from those seen in Type I or Type II αMNs, in which not only the nucleus but also the cytoplasm were invariably immunopositive for NeuN (**Figures 1**, **3**) Around the recorded neuron in this frame examined (**Figure 5F**), there were ChAT(+) and NeuN(+/N, +/C) αMNs (filled arrowheads) or ChAT(+) and NeuN(+/N, -/C) MNs but non-αMNs (open arrowheads). This pattern was similar to the observation in **Figure 1F** but in contrast to that in **Figure 3F**. Taking these observations into consideration together with the postnatal downregulation of NeuN expression in γMNs in contrast to the prenatal upregulation of NeuN in αMNs (Shneider et al., 2009), we classified the both NeuN(-/N, -/C) and NeuN(+/N, -/C) MNs as γMNs (see section “Discussion”). Indeed, there was no significant (^∗^*p* > 0.8) difference in the mean size between NeuN(-/N, -/C) MNs (19 ± 3 μm; n = 21) and NeuN(+/N, -/C) MNs (19 ± 1 μm; n = 24) whereas both the NeuN(-/N, -/C) and NeuN(+/N, -/C) MNs were significantly (^∗^*p* < 0.004 and ^∗^*p* < 0.003, respectively) smaller than Type I αMNs (28 ± 2 μm; n = 19). These findings suggest that small-sized MNs that display the pulse-ADP together with the persistent inward current are γMNs.

### Ionic Mechanisms Underlying Pulse-ADP

**FIGURE 6:**
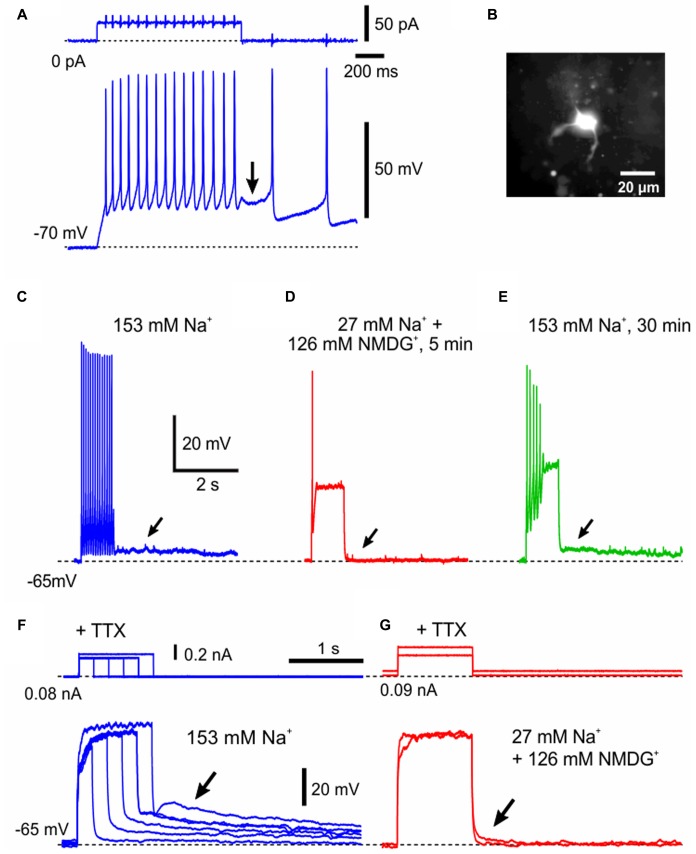
The generation of pulse-ADP is dependent on extracellular Na^+^. **(A)** An application of a depolarizing current pulse in a neuron at the resting membrane potential of –70 mV caused a spike train, which was followed by a pulse-ADP (arrow) that triggered further spikes. **(B)** A lucifer yellow image of the recorded neuron classified as γMN, which displayed sparse arborizations of primary dendrites. **(C)** A spike train that was followed by a pulse-ADP lasting for more than 5 s (arrow) was induced in aCSF containing 153 mM Na^+^. **(D)** Abolishment of the pulse-ADP (**C**, arrow) by substitution of 126 mM Na^+^ with the equimolar NMDG^+^. **(E)** Recovery of the pulse ADP (arrow) after washout of NMDG^+^ with the original aCSF. Compare **(E)** with **(C,D)**. **(F)** In the presence of 1 μM TTX, the amplitude of pulse-ADPs increased (arrow) as the duration or the amplitude of the depolarizing current pulse increased. **(G)** In the presence of TTX, the pulse-ADP was abolished (arrow) by substitution of 126 mM Na^+^ with the equimolar NMDG^+^.

The pulse-ADP can occasionally trigger spike firings (**Figure 6A**) as was the case with that recorded in an MN (**Figure 6B**), and often lasted for more than 5 s (**Figure 6C**). In such an MN that displayed a long-lasting pulse-ADP, extracellular Na^+^ was substituted with NMDG^+^ to investigate the ionic mechanism underlying the pulse-ADP. When 126 mM Na^+^ contained in the aCSF was substituted with equimolar NMDG^+^, the pulse-ADP was almost completely abolished although the generation of action potentials was largely suppressed (**Figures 6C,D**). After perfusion with the original aCSF, the pulse-ADP was restored (**Figure 6E**). Furthermore, in the presence of 1 μM TTX that blocks voltage-dependent Na^+^ channels, pulse-ADPs were induced by injecting depolarizing current pulses, and the amplitude of pulse-ADP increased as the duration or the amplitude of the depolarizing current pulse was increased (**Figure 6F**). Consistent with the abolishment of the pulse-ADP following action potentials by Na^+^ substitution with NMDG^+^ (**Figures 6C–E**), the pulse-ADP following depolarization evoked in the presence of TTX was also abolished by Na^+^ substitution with NMDG^+^ (**Figure 6G**). These findings indicate that the more depolarization, the more generation of the pulse-ADP, regardless of the presence or absence of Na^+^ action potentials. These results suggest pulse-ADP may be mediated by Ca^2+^-dependent cation channels (Kang et al., 1998). This possibility was examined in the next experiment.

**FIGURE 7:**
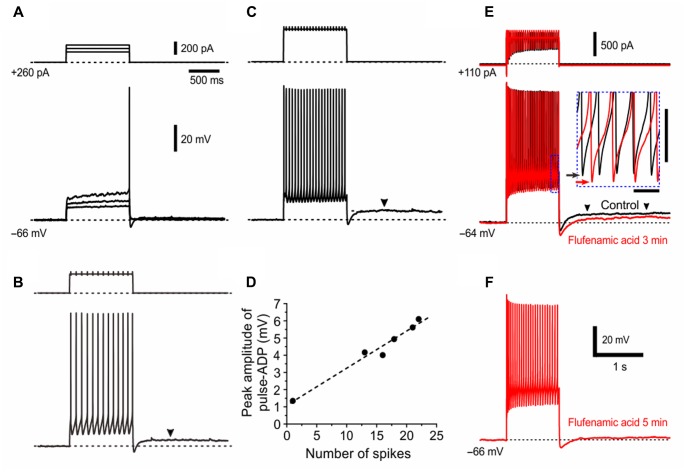
The pulse-ADP presumably mediated by Ca^2+^-dependent cation channels. **(A–C)** Membrane potential responses to depolarizing current pulses applied in a presumed γMN at –66 mV which was brought about by a positive DC current injection of 260 pA. The amplitudes of pulse-ADPs increased with increases in the number of spikes. Arrowheads indicate pulse-ADPs **(B,C)**. Also note a superlinear *I–V* relationship of the subthreshold responses in **(A)**, similar to **Figure 5B**. **(D)** A linear relationship between the number of spikes and the peak amplitude of pulse-ADP. **(E,F)** Effects of flufenamic acid on the pulse-ADP induced following a train of spikes evoked by a constant depolarizing current pulse applied in a presumed γMN at –64 mV, examined before (**E**, black) and 3 min (**E**, red) and 5 min after application of 10 μM flufenamic acid **(F)**. Arrowheads indicate pulse-ADPs **(E)**. The inset in **(E)** shows the traces included in an area enclosed by a rectangle (blue dotted line) on the expanded time and voltage scales. Black and red arrows indicate the most negative levels of the afterhyperpolarization obtained before (**E**, black) and 3 min after application of flufenamic acid (**E**, red). Time and voltage calibrations for inset; 50 ms and 10 mV, respectively. Time and voltage calibrations in **(F)** apply also in **(E)**.

Consistent with the hypothesized involvement of Ca^2+^-dependent cation channels, the amplitude of pulse-ADP increased with an increase in the number of spikes that can activate Ca^2+^ channels (**Figures 7A–C**), as revealed by a linear relationship between number of spikes and the peak amplitude of pulse-ADP (**Figure 7D**). Six γMNs consistently displayed this relationship. To directly clarify whether the pulse-ADP is mediated by Ca^2+^-dependent cation channels, we examined the effects of flufenamic acid, a Ca^2+^-dependent cation channel blocker, on pulse-ADPs. Three min after application of flufenamic acid at 10 mM, the amplitude of pulse-ADPs was decreased by 43% (**Figure 7E**). In six γMNs, bath application of flufenamic acid for 5 min significantly (^∗∗^*p* < 0.001) decreased the amplitude of pulse-ADPs from 3.5 ± 1.2 to 0.70 ± 1.3 mV by 84 ± 25% but did not significantly (^∗∗^*p* > 0.8) decrease the number of spikes from 30.2 ± 6.0 to 29.7 ± 7.0 (**Figure 7F**). These results strongly suggest that pulse-ADP is mediated by Ca^2+^-dependent cation channels. It is noteworthy that the bottom peak level of afterhyperpolarization was lowered by flufenamic acid in association with the decrease in the amplitude of pulse-ADP (**Figure 7E**, inset), suggesting that the responsible current for the generation of the pulse-ADP is already active during repetitive spiking.

### Morphological and Electrophysiological Differences among Type I and II αMNs and γMNs

**FIGURE 8:**
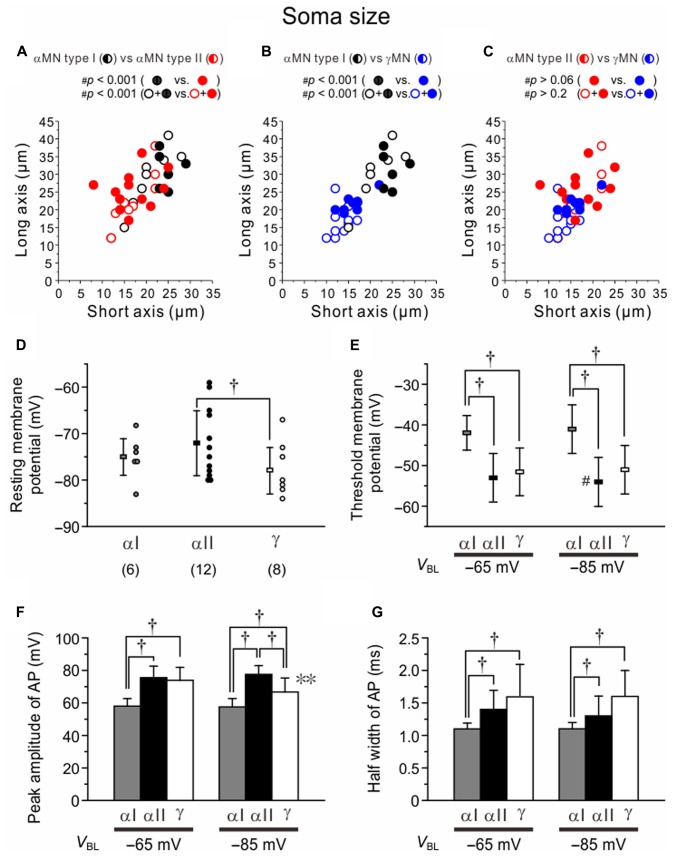
Differences in the soma size and electrophysiological property among Type I and II αMNs and γMNs. **(A–C)** Differences in the distribution profiles of short and long axis sizes of somata in the two-dimensional space between Type I and II αMNs **(A)**, between Type I αMNs and γMNs **(B)**, and between Type II αMNs and γMNs **(C)**. Black, red, and blue filled circles represent Type I and Type II αMNs and γMNs, respectively, which were classified both electrophysiologically and immunohistochemically. Black, red, and blue open circles represent Type I and Type II αMNs and γMNs, respectively, which were classified only electrophysiologically. Half-filled circles represent both filled and open circles of the same color. **(D)** The resting membrane potentials in Type I and II αMNs and γMNs. ^†^*p* < 0.05. **(E)** The threshold membrane potentials of spikes evoked at –65 and –85 mV in Type I and II αMNs and γMNs. ^†^*p* < 0.02. **(F)** The peak amplitudes of action potentials evoked at –65 and –85 mV in Type I and II αMNs and γMNs. ^†^*p* < 0.05. ^∗∗^*p* < 0.05. **(G)** The half-width values of action potentials evoked at –65 and –85 mV in Type I and II αMNs and γMNs. ^†^*p* < 0.03.

There were significant differences in the distribution profile of short and long axis sizes of somata in the two-dimensional space between Type I (black) and II (red) αMNs (**Figure 8A**). When the comparison was made between the Type I (*n* = 14) and Type II αMNs (*n* = 22) that were classified based on the electrophysiological observations *both* with (black and red filled circles; *n* = 6 and 12, respectively) and without IH identification (black and red open circles; *n* = 8 and 10, respectively), ^#^*p*-value was < 0.001. When compared between the two types of αMNs classified based on the electrophysiological observations *solely* with IH identification (black and red filled circles), ^#^*p*-value was < 0.001. There were also significant differences in the distribution of soma sizes between Type I αMNs (black) and γMNs (blue) that were classified electrophysiologically *both* with and without IH identification (*n* = 14 and 18, respectively, ^#^*p* < 0.001) or electrophysiologically *solely* with IH identification (*n* = 6 and 8, respectively, ^#^*p* < 0.001) (**Figure 8B**). However, there was no significant difference in the distribution of soma sizes between Type II αMNs (red) and γMNs (blue) that were classified electrophysiologically *both* with and without IH identification (*n* = 22 and 18, respectively, ^#^*p* > 0.06) or electrophysiologically *solely* with IH identification (*n* = 12 and 8, respectively, ^#^*p* > 0.2) (**Figure 8C**). The mean size of the soma was significantly smaller (^∗^*p* < 0.001) in Type II (20 ± 4 μM) than in Type I αMNs (28 ± 2 μM). Consistent with this finding, the input resistance was significantly (^∗^*p* < 0.004) higher in Type II (212 ± 122 MΩ) than in Type I αMNs (75 ± 16 MΩ).

The resting membrane potentials in Type I and II αMNs and γMNs were -75 ± 4 mV (*n* = 6), -72 ± 7 mV (*n* = 12) and -78 ± 5 mV (*n* = 8), respectively (**Figure 8D**). There was significant (^†^*p* < 0.05) difference in the resting membrane potential between Type II αMNs and γMNs (**Figure 8D**). When action potentials were evoked at -65 mV, the threshold membrane potential in Type I αMNs (-42 ± 4 mV) was significantly more positive than those in Type II αMNs (-52 ± 6 mV, ^†^*p* < 0.003) and γMNs (-52 ± 6 mV, ^†^*p* < 0.006) (**Figure 8E**). When action potentials were evoked at -85 mV, the threshold membrane potential in Type I αMNs (-41 ± 6 mV) was also significantly more positive than those in Type II αMNs (-54 ± 6 mV, ^†^*p* < 0.004) and γMNs (-51 ± 6 mV, ^†^*p* < 0.02) (**Figure 8E**). These results suggest that the higher threshold membrane potential is due to *I*_KA_. However, in Type I αMNs, the threshold membrane potential at -65 mV was not significantly (^∗∗^*p* > 0.3) different from that at -85 mV (**Figure 8E**). This suggests that voltage-dependent inactivation of *I*_KA_ is not prominent at -65 mV. Furthermore, in γMNs, the threshold membrane potential at -65 mV was not significantly (^∗∗^*p* > 0.07) different from that at -85 mV (**Figure 8E**). In contrast, in Type II αMNs, the threshold membrane potential at -85 mV was significantly (^∗∗^*p* < 0.007) more negative than that at -65 mV (**Figure 8E**). This result may be due to activation of LTS.

The peak amplitude of action potentials evoked at -65 mV in Type I αMNs (58 ± 5 mV) was significantly smaller than those in Type II αMNs (76 ± 7 mV, ^†^*p* < 0.001) and γMNs (73 ± 8 mV, ^†^*p* < 0.002) (**Figure 8F**). These results would reflect that *I*_KA_ increases the threshold membrane potentials and facilitates spike repolarization. The peak amplitudes of action potentials evoked at -85 mV in Type I αMNs (58 ± 5 mV) and γMNs (67 ± 8 mV) were significantly (^†^*p* < 0.001 and ^†^*p* < 0.02, respectively) smaller than that in Type II αMNs (78 ± 8 mV), and that in Type I αMNs was significantly (^†^*p* < 0.05) smaller than that in γMNs (**Figure 8F**). The peak amplitude of action potentials evoked at -85 mV in γMNs was significantly (^∗∗^*p* < 0.05) smaller than that evoked at -65 mV while the peak amplitudes of action potentials evoked at -85 mV in Type I αMNs and Type II MNs were not significantly (^∗∗^*p* > 0.6 and ^∗∗^*p* > 0.2, respectively) different from those evoked at -65 mV in Type I αMNs and Type II αMNs (**Figure 8F**). The half width of action potentials evoked at -65 mV in Type I αMNs (1.1 ± 0.1 ms) was significantly (^†^*p* < 0.02 and ^†^*p* < 0.03, respectively) shorter than those in Type II αMNs (1.4 ± 0.3 ms) and γMNs (1.6 ± 0.5 ms), and the half width of action potentials evoked at -85 mV in Type I αMNs (1.1 ± 0.1 ms) was also significantly (^†^*p* < 0.02 and ^†^*p* < 0.02, respectively) shorter than those in Type II αMNs (1.3 ± 0.3 ms) and γMNs (1.6 ± 0.4 ms) (**Figure 8G**). There results would reflect mostly the activity of *I*_KA_ in Type I αMNs.

## Discussion

As summarized in **Table 1**, we found that in the dl-TMN NeuN-positive αMNs were divided into two subtypes, Type I and Type II αMNs: Type I αMNs had a relatively larger cell body and displayed a 4-AP-sensitive K^+^ current while Type II αMNs had a relatively smaller cell body and displayed LTS and a less prominent 4-AP sensitive K^+^ current. The presence of two types of αMNs in the TMN found in the present study using infant rats at PND 7–12 was consistent with the observations made in the TMN of juvenile guinea pigs or adult rats, in which MNs displayed either 4-AP sensitive delayed spiking (Chandler et al., 1994) or Ni^2+^ sensitive LTS (Kobayashi et al., 1997). We also found that the MNs which were either NeuN(-/N, -/C) or NeuN(+/N, -/C) both equally had smaller cell bodies and both equally displayed a characteristic pulse-ADP mediated by flufenamate-sensitive Ca^2+^-dependent cation current and persistent inward current (*n* = 4 and 4, respectively). These MNs are most likely to be γMNs, in view of the postnatal downregulation of NeuN expression in γMNs (Shneider et al., 2009). In addition, there were small-sized non-cholinergic neurons, presumably GABAergic interneurons, which displayed a prominent LTS but not *I*_KA_, pulse-ADPs and persistent inward current (**Table 1**). The size distribution profile of Type I and II αMNs and γMNs found in the present study was consistent with our previous result (Morita-Isogai et al., 2017).

**Table 1:** Electrophysiological classification of neurons and their sizes in the dl-TMN.

	αMN						
	Type I		Type II		γMN		Non-MN
	Total	ChAT(+) NeuN(+/N, +/C)	Total	ChAT(+) NeuN(+/N, +/C)	Total	ChAT(+) NeuN(±/N, -/C)	ChAT(-) NeuN(+)
Number of cells	14	6	22	12	18	8	11
Soma size (μm)							
Long axis	30 ± 7	31 ± 5	24 ± 7	26 ± 6	19 ± 4	22 ± 2	12 ± 4
Short axis	23 ± 4	25 ± 3	17 ± 4	17 ± 5	15 ± 3	16 ± 3	12 ± 3
4-AP-sensitive K^+^ current (transient *I*_KA_)	+		+		-		-
Low-threshold spike (LTS)	-		+		-		+
Pulse afterdepolarization (pulse-ADP)	-		-		+		-
Persistent inward current	-		-		+		-

Furthermore, we found that in Type I αMNs (*n* = 8) the slope of the *I*–*F* plot for the first IF was significantly smaller than that for the SS-F (**Figure 2E**) whereas in Type II αMNs (*n* = 11) the slope for the first IF was significantly larger than that for the SS-F (**Figure 4E**). There results would reflect the activity of *I*_KA_ in Type I αMNs and the activity of LTS in Type II αMNs at the onset of current pulses. Similar differences in the firing pattern were also observed between a larger and smaller MN in the dl-TMN in our previous study showing the size-based orderly recruitment of MNs in the dl-TMN (see **Figures 3**, **6**; Okamoto et al., 2016). The slope of the *I*–*F* plot for the first IF in Type I αMNs was significantly (^†^*p* < 0.02 and ^†^*p* < 0.001, respectively) smaller than that in Type II αMNs, which in turn was significantly (^†^*p* < 0.002) smaller than that in γMNs. Although there was no significant (^†^*p* > 0.6) difference in the slope of the *I*–*F* plot for the SS-F between Type I and II αMNs, the slopes of the *I*–*F* plot for the SS-F in Type I and II αMNs were significantly (^†^*p* < 0.001 and ^†^*p* < 0.001, respectively) smaller than that in γMNs. These results suggest that the intrinsic excitability in ascending order is: Type I αMNs < Type II αMNs < γMNs.

### Development of αMNs

The electrophysiological classification of MNs in the dl-TMN of rats at PND 7–12 based on the presence of different subsets of currents was consistent with that studied in juvenile guinea pigs (Chandler et al., 1994) although the frequency of encountering such MNs that display 4-AP sensitive delayed spiking was very low compared to the present study. A similar classification of αMNs based on A-type K^+^ current and T-type Ca^2+^ current has been reported in rat hypoglossal MNs (Viana et al., 1995) and abducens MNs (Russier et al., 2003). However, in these motor nuclei, such MNs that display 4-AP sensitive currents temporarily existed only during an early postnatal period and disappeared after that. In intracellular recordings from rat trigeminal MNs at 3–6 weeks of age, 89% of MNs showed spike-ADP partly composed of Ni^2+^ sensitive LTS while 11% of MNs did not show spike-ADP (Kobayashi et al., 1997). Considering the difficulty in blindly encountering the smallest γMN with sharp microelectrodes, those MNs not showing spike-ADP may be the other type of αMNs. In the previous two studies in TMN (Chandler et al., 1994; Kobayashi et al., 1997), action potentials were evoked at the resting membrane potential (-62 and -67 mV, respectively), at which 4-AP sensitive K^+^ current was largely inactivated (half-inactivation voltage = -80 mV; Matsuo and Kang, 1998), and the delayed spiking would be masked unless the baseline membrane potential is hyperpolarized. This can be the reason why there were only small numbers of MNs that showed delayed spiking. The small number of the MNs displaying A-type K^+^ currents could also be due to the low viability of those MNs in brain slice preparations. Nevertheless, it is not certain or conclusive that Type I αMNs keep existing in TMN of adult animals. However, provided that Type I αMNs were present in TMN of adult animals, the tension of jaw-closing muscles could be finely tuned over a wide range during occlusion, due to the presence of Type I delayed spiking αMNs in addition to Type II αMNs in the dl-TMN. To our best knowledge, there has been no report showing that rank-ordered recruitment is involved in the control of eye or tongue movement, probably because any isometric contraction does not take place in the tongue and lateral rectus muscles. This may be the reason why in the hypoglossal or abducens motor nucleus such MNs that display 4-AP sensitive currents do not exist in adult, given the persistent presence of Type I delayed spiking αMNs in TMN.

### Development of γMNs

It has been demonstrated that ∼30% of γMNs are weakly immunopositive for NeuN especially in their nuclei during early postnatal periods to PND 20 while 100% of αMNs are strongly immunopositive for NeuN both in the nucleus and cytoplasm at PND 0 (Shneider et al., 2009). On the other hand, Err3 was expressed by most MNs in the early postnatal stages and the selective expression of Err3 in γMNs gradually occurred over the first two postnatal weeks (Friese et al., 2009). Thus, after the first two postnatal weeks, αMNs and γMNs in spinal MNs become molecularly distinguishable by the differential expression of NeuN and Err3 (Friese et al., 2009). In αMNs, NeuN has already been upregulated to be expressed not only in nucleus but also in cytoplasm at birth whereas Err3 is downregulated along with postnatal development. In contrast, in γMNs, NeuN is downregulated along with postnatal development whereas Err3 is maintained. In the present study performed using rats at PND 7–12, consistent with these reports, almost all MNs were Err3-positive (figure not shown) while MNs displayed three different immunoreactivities to NeuN. Therefore, classification of MNs was made based on the immunoreactivity to NeuN. In accordance with the previous reports (Friese et al., 2009; Shneider et al., 2009; Morita-Isogai et al., 2017), the first type of MNs that showed the prominent immunoreactivity for NeuN not only in their nucleus but also in their cytoplasm were classified as αMNs, and the second type of MNs that showed no immunoreactivity for NeuN in the nucleus and cytoplasm, namely NeuN(-/N, -/C) MNs were classified as γMNs (**Figure 3**). The third type of MNs that showed the relatively weak immunoreactivity for NeuN only in nucleus but not in cytoplasm, namely NeuN(+/N, -/C) MNs were also classified as γMNs (**Figure 5**), also in accordance with the previous report (Shneider et al., 2009). Regardless of Type I or II, αMNs were invariably immunopositive for NeuN both in the nucleus and cytoplasm. As proposed previously (Ashrafi et al., 2012; Enjin et al., 2012), wnt7a or serotonin receptor 1d may be necessary to further confirm the electrophysiological properties of γMNs.

In rats, the transition from suckling to chewing occurs around PND 12, and the mature mastication pattern is acquired around postnatal weeks 2–3 (Thexton and Griffiths, 1979; Westneat and Hall, 1992). Considering that γMNs play a crucial functional role in the isometric contraction during chewing foods (Tsukiboshi et al., 2012), it is possible that the development of γMNs is closely associated with the transition from suckling to chewing. If this is the case, the downregulation of Err3 expression selectively in αMNs and that of NeuN expression selectively in γMNs of the dl-TMN would be completed around postnatal weeks 2–3, at which ages patch-clamp recordings of MNs in brainstem slices are hardly possible because of much less viability of MNs due to severance of many dendrites of MNs extending every direction.

### Ionic Mechanism for Pulse-ADP and its Functional Implications

Pulse-ADP was found to be mediated by a cationic current as demonstrated by the experiment in which extracellular Na^+^ was substituted by NMDG^+^ (**Figures 6C–E**). There are several types of cationic currents with a similar ionic selectivity, which are either activated Ca^2+^ dependently (Kang et al., 1998) or independently (Alzheimer, 1994) or through activation of G-protein coupled receptors (Guerineau et al., 1995; Fraser and MacVicar, 1996). More recently, TRPC channels were identified to be responsible for the ADP induced by activation of muscarinic receptors in cortical pyramidal cells (Yan et al., 2009), and it has been reported that ADP-induced bursting was blocked by flufenamic acid in respiratory MNs (Pace et al., 2007) or in cortical pyramidal cells (Lin et al., 1998). Therefore, it is possible that the pulse-ADP found in γMNs may also be enhanced by activation of some metabotropic receptors. Once γMNs are activated, the long-lasting ADP may cause a “tonic-like firing” when metabotropic receptors are activated simultaneously. This long-lasting tonic drive of Ia synaptic inputs by γMNs is especially important for TMN αMNs. This is because the number of synapses between single Ia afferents and single TMN αMNs is much smaller (Dessem and Taylor, 1989; Yabuta et al., 1996) compared to the Ia-spinal αMN synapses (Redman and Walmsley, 1983a,b), and because single γMNs may innervate a larger number of intrafusal fibers contained in single muscle spindles (Eriksson et al., 1994), compared to the limb motor system (Sahgal et al., 1985). Indeed, in limb muscles, the spatial summation of Ia-excitatory postsynaptic potentials (EPSPs) would easily activate αMNs, while in masseter muscles, the temporal summation of Ia-EPSPs would be required to activate αMNs as reflected in the failure and success in evoking H-reflex in the resting and the slight clenching condition of masseter muscles, respectively (Fujii and Mitani, 1973).

### Functional Implication of Morphological and Electrophysiological Differences between Type I and II αMNs in the Orderly Recruitment of MNs

The mean size of the soma was significantly smaller in Type II than in Type I αMNs, consistent with the higher input resistance of Type II compared to Type I αMNs. Furthermore, the threshold for activation of spikes in Type II αMNs was also significantly lower than that in Type I αMNs, not only due to the higher input resistance but also due to the presence of LTS which is presumably mediated by Ni^2+^-sensitive inactivating Ca^2+^ current as reported previously in the same TMN (Kobayashi et al., 1997). Thus, the soma size, the input resistance and the spike threshold were in favor of the orderly recruitment of αMNs from Type II to Type I. Furthermore, the fast phasic firing followed by tonic firing in Type II αMNs was in contrast to the delayed tonic firing in Type I αMNs. Thus, given the persistent presence of Type I αMNs, these differences in the intrinsic membrane property and the subsequent firing pattern between the two types of TMNs may be the bases for the rank-ordered recruitment of TMNs, although the group Ia synaptic input is also known to be in favor of the rank-ordered recruitment of spinal MNs (Heckman and Binder, 1993). Although we did not measure the spike afterhyperpolarization, the spike duration was also significantly larger in Type II than in Type I αMNs, consistent with the classical classification of slow and fast MNs (Eccles et al., 1957, 1958).

The occlusal phase of mastication cycle especially requires a very fine isometric contraction tuning over a wide range; in a lower range as fine as the tone tuning of lumbrical muscles while in a higher range as strong as that of hand muscles. In the isometric contraction, spindle Ia activity caused by the activity of γMNs can be a gain controller (Greer and Stein, 1990) of synaptic inputs arising from premotor neurons or the pattern generator onto αMNs. Then, a wide range tuning of isometric contraction can be achieved by tuning the characteristic tonic activity of γMNs, which is mediated by Ca^2+^-dependent cationic current. We have also previously shown that the ratio of number of γMNs to all MNs in the dl-TMN (Morita-Isogai et al., 2017) is 17% higher than that in the spinal motor neuron pool (Friese et al., 2009). Thus, the TMN is specialized by the presence of presumably the two types of αMNs and by the larger ratio of the number of γMNs to that of all MNs to achieve the wide range fine tuning. These specializations would not be seen in other limb motor system.

## Author Contributions

YK designed the experiments. YK and HT wrote the manuscript. KN, MO, MS, YM-I, HS, EK, and DY performed the research. All authors analyzed the data.

## Conflict of Interest Statement

The authors declare that the research was conducted in the absence of any commercial or financial relationships that could be construed as a potential conflict of interest.

## Abbreviations

αMN: α-motoneuron
γMN: γ-motoneuron
4-AP: 4-aminopyridine
aCSF: artificial cerebrospinal fluid
ADP: afterdepolarization
dl-TMN: dorsolateral trigeminal motor nucleus
EPSP: excitatory postsynaptic potential
Err3: estrogen-related receptor gamma
IF: instantaneous frequency
IH: immunohistochemical
*I*_KA_: A-type K^+^ current
LTS: low-threshold spike
PND: postnatal day
SS: steady state
